# Comment on “Factors Affecting the Effectiveness of Hospital Incident Command System; Findings from a Systematic Review”

**DOI:** 10.30476/beat.2020.86250

**Published:** 2020-10

**Authors:** MohammadBagher Shamsi, Zeinab Rahimzadeh Sani, Maryam Mirzaei

**Affiliations:** 1 *School of Allied Medical Sciences Kermanshah University of Medical Sciences, Kermanshah, Iran*; 2 *Department of Food Science and Technology, Faculty of Nutrition, Tabriz University of Medical Sciences, Tabriz, Iran*; 3 *Department of Rehabilitation and Sports Medicine, School of Allied Medical Sciences, Kermanshah University of Medical Sciences, Kermanshah, Iran*

***Dear Editor,***

Bahrami *et al*. published an interesting article entitled "Factors Affecting the Effectiveness of Hospital Incident Command System; Findings from a Systematic Review” [1]; it seems that this systematic review, as many other review published, do not pay proper attention to the reporting: 

1. Data Sources 

Springer which is an academic publisher and it could be considered as the sources to search, therefore, should not be included in searched databases. Again, Google Scholar is a search engine. It will be better for researchers to specify which one is a database and which one is a publisher, search engine and etc [2].

2. Evidences Quality Assessment

The researchers have evaluated the selected evidences from methodological aspects by a 7-question checklist, but there is no information about the developing and accessing of validity and reliability of this checklist. In addition, the quality assessment summary was addressed in the method section. This item should be clearly reported in the results [2].

3. Results Presentation 

According to preferred reporting items for systematic review and meta-analysis (PRISMA) checklist, the results presentation of search strategy should be presented as a subsection of the results [2, 3]. In this review, the PRISMA flow diagram is addressed in the methods section; that is incorrect. 

4. Registration 

The final point is about the importance of the registering the review; Due to substantial increase in the quantity of systematic reviews in the resent years, it is important to review protocols to be registered before it is conducted/initiated (PROSPERO database: International prospective register of systematic reviews, http://www.crd.york.ac.uk/prospero). This is a good practice to avoid unplanned duplication of systematic reviews and to show readers that researcher have adhered to their protocol [2].

## Conﬂicts of interest

 There are no conﬂicts of interest.
